# Can a Single Measurement of Apixaban Levels Identify Patients at Risk of Overexposure? A Prospective Cohort Study

**DOI:** 10.1055/s-0041-1740492

**Published:** 2022-01-24

**Authors:** Tim A.C. de Vries, Jack Hirsh, Vinai C. Bhagirath, Jeffrey S. Ginsberg, Ron Pisters, Martin E.W. Hemels, Joris R. de Groot, John W. Eikelboom, Noel C. Chan

**Affiliations:** 1 Department of Cardiology, Amsterdam University Medical Centers, Location Academic Medical Center, Amsterdam, North Holland, The Netherlands; 2 Department of Cardiology, Rijnstate Hospital, Arnhem, Gelderland, The Netherlands; 3 Thrombosis and Atherosclerosis Research Institute, McMaster University, Hamilton, Ontario, Canada; 4 Department of Medicine, McMaster University, Hamilton, Ontario, Canada; 5 Department of Cardiology, Radboud University Medical Centre, Nijmegen, Gelderland, The Netherlands; 6 Population Health Research Institute, McMaster University, Hamilton, Ontario, Canada

**Keywords:** Biological variation, individual, Biological variation, population, Blood coagulation tests, Drug monitoring, Factor Xa inhibitors, Off-label use

## Abstract

**Background** Patients with atrial fibrillation (AF) are frequently treated with apixaban 2.5-mg twice daily (BID) off-label, presumably to reduce the bleeding risk. However, this approach has the potential to increase the risk of ischemic stroke. If a single measurement could reliably identify patients with high drug levels, the increased stroke risk may be mitigated by confining off-label dose reduction to such patients.

**Objectives** This study aimed to determine whether a single high apixaban level is predictive of a similarly high level when the test is repeated in 2 months.

**Methods** In this prospective cohort study of clinic patients receiving apixaban 5-mg BID for AF or venous thromboembolism, peak and trough apixaban levels were measured using the STA-Liquid anti-Xa assay at baseline and 2 months. We calculated the proportions of patients with levels that remained in the upper quintile.

**Results** Of 100 enrolled patients, 82 came for a second visit, 55 of whom were treated with apixaban 5-mg BID. Seven (63.6%, 95% confidence interval [CI]: 35.4–84.8%) and nine (81.8%, 95% CI: 52.3–94.9%) of 11 patients with a baseline trough and peak level in the upper quintile, respectively, had a subsequent level that remained within this range. Only one (9.1%, 95% CI: 1.6–37.7%) patient had a subsequent level that fell just lower than the median.

**Conclusion** The trough and peak levels of apixaban in patients who have a high level on a single occasion, usually remain high when the assay is repeated in 2 months. Accordingly, the finding of a high apixaban level in patients deemed to be at high risk of bleeding, allows physicians contemplating off-label use of the 2.5-mg BID dose to limit its use to selected patients who are less likely to be exposed to an increased risk of thrombosis.

## Introduction

Direct-acting oral anticoagulants (DOACs) have revolutionized oral anticoagulant therapy because they were shown to be safe and effective without the need for routine monitoring of their anticoagulant activity. 1 Apixaban, the third DOAC to be approved, was found to be more effective and safer than warfarin for stroke prevention in patients with atrial fibrillation (AF). 2 3 4

In the randomized trials that led to its approval, apixaban was given in a standard dose of 5-mg twice daily (BID) in most patients and in a reduced dose of 2.5-mg BID in patients who met at least two of three “ABC” criteria (age ≥80 years, body weight ≤ 60 kg, and serum creatinine ≥133 µmol/L). 3 5 Accordingly, 2.5-mg BID is recommended for patients who meet these criteria. 4 6 7

Despite these recommendations, in daily practice approximately 50% of patients are treated with the 2.5-mg dose without meeting the labeled criteria for dose reduction (i.e., “off-label” dose reduction), 8 9 presumably because treating physicians are concerned with the risk of bleeding. This is understandable because a substantial number of patients treated in clinical practice have higher bleeding risks than those included in clinical trials. 9 10 Yet, many of the same patients are also at a higher risk of ischemic stroke, 9 10 and as a consequence, several authors have criticized the off-label use of the reduced dose. 8 11 12 13 14

We postulate that, decision-making on the risk benefit ratio of lowering the dose of apixaban from 5- to 2.5-mg BID in selected high-risk AF patients might be facilitated by performing limited drug monitoring. 15 16 Thus, physicians who plan to reduce the dose of apixaban in patients deemed at high risk of bleeding on clinical grounds, might be reassured if they could limit dose reduction to those who have consistently high drug levels. The inconvenience of such an approach would be small if a single drug level was predictive of sustained high levels.

As a first step in investigating whether limited dose adjustment based on a single drug level measurement would be clinically worthwhile, we performed a study to determine whether a high drug level measured on one occasion is sustained over the next 2 months.

## Methods

### Manuscript and Study Design

This manuscript follows the guidelines of the Strengthening the Reporting of Observational studies in Epidemiology (STROBE) statement ( Supplementary Table S1 ). 17 We performed a prospective cohort study of adult (defined as ≥18 years) patients taking either 2.5- or 5-mg BID of apixaban for stroke prevention in AF or for treatment or secondary prevention of venous thromboembolism (VTE). This study was designed to answer questions on the variability of apixaban levels between patients (i.e., interpatient variability), as well as on the variability within patients (i.e., intrapatient variability). As this was a noninterventional study, the treating physicians were unaware of the results of the drug level measurements. The study protocol was reviewed and approved by the Hamilton Integrated Research Ethics Board, and patients were enrolled between October 2014 and May 2017. None of the data reported here have been published previously.

**Table 1:** Patient characteristics

	All ( *n* = 82)	Apixaban 5-mg BID ( *n* = 55)
Apixaban dose		
5-mg BID	55 (67.1)	55 (100.0)
2.5-mg BID	27 (32.9)	0 (0.0)
Indication		
Atrial fibrillation	69 (84.1)	50 (90.9)
Venous thromboembolism	13 (15.9)	5 (9.1)
Number of ABC criteria		
0	57 (69.5)	39 (70.9)
1	21 (25.6)	15 (27.3)
≥2	4 (4.9)	1 (1.8)
Age ^a^ (y)	73.1 (66.1–79.8)	72.4 (66.5–78.7)
Male	46 (56.1)	35 (63.6)
Weight (kg)	82.9 ± 16.7	84.5 ± 15.5
BMI ^a^ (kg/m ^2^ )	28.3 (25.6–31.5)	28.4 (26.0–31.7)
CrCl ^a^ (mL/min)	80.6 (61.1–101.2)	82.5 (59.9–100.6)
History of heart failure	15 (18.3)	11 (20.0)
Alcohol use	21 (25.6)	16 (29.1)
Ongoing smoking habit	4 (4.9)	1 (1.8)
P-gp and/or CYP3A4 inhibitor	11 (13.4)	9 (16.4)
Amiodarone	6 (7.3)	6 (10.9)
Diltiazem	5 (6.1)	3 (5.5)
P-gp and/or CYP3A4 inducer	0 (0.0)	0 (0.0)

Abbreviations: ABC, age, body weight, and serum creatinine; BID, twice daily; BMI, body mass index; CrCl, Creatinine Clearance; CYP3A4, Cytochrome P450 3A4; P-gp, P-glycoprotein.

Note: Continuous data are reported as mean±standard deviation if normally distributed, and as median (interquartile range) ^a^ if not. Categorical data are in number (percentage).

### Patients

Consecutive eligible clinic patients visiting the Hamilton General Hospital were asked to participate. Patients were eligible if they were treated with apixaban and had received at least 1 week of treatment. Those who were either geographically inaccessible for follow-up, or unwilling or unable to provide written informed consent were not eligible for inclusion. The final sample size of 100 patients was chosen based on feasibility of recruitment.

### Collection, Processing, and Analyses of Blood Samples

We measured both trough and peak levels at the first visit (i.e., baseline) and at 2 months ± 2 weeks (i.e., follow-up). On both occasions, a blood sample was taken before the morning dose and 3 hours after administration of apixaban, for trough and peak apixaban levels, respectively. At each time point, 10 mL of blood was collected into Becton Dickinson Vacutainer tubes (Becton Dickinson, Mississauga, Ontario, Canada), containing 3.2% buffered trisodium citrate (9:1, vol/vol) by a trained research assistant. Immediately after collection, the tube was inverted three to five times, cellular elements were sedimented by twice subjecting the sample to centrifugation at 1,700 g for 15 minutes at 23°C. The resultant platelet-poor plasma was then harvested and stored in 1-mL aliquots at −80°C. Apixaban concentrations were measured using the STA-Liquid anti-Xa assay with high-performance liquid chromatography referenced apixaban calibrators and controls using a Diagnostica Stago STA-R Evolution automated analyzer (Diagnostica Stago, Asnieres-Sur-Seines, France) according to manufacturer's recommendations. 18 19 Apixaban concentrations measured by our apixaban-calibrated anti-Xa method have excellent agreement with plasma apixaban concentrations as measured by liquid chromatography/tandem mass spectrometry. 20 21 Further, our assay outputs results in ng/mL, and we therefore refer to our results as drug levels which is consistent with previous reports. 22 23 24 25 The lower limit of detection for this assay is 20 ng/mL. Any levels that were below the level of detection were given a value half the assay limit (i.e., 10 ng/mL).

Clinical characteristics (e.g., age, body weight, serum creatinine, and concurrent medication) were recorded at baseline, and any change in medication at the second visit. Consistent with the Summary of Product Characteristics of apixaban, 4 6 we defined off-label use according to the ABC criteria only in patients with AF but estimated the number of present ABC criteria in all patients.

### Statistical Analyses

Continuous data were summarized using means and standard deviations (SDs) if normally distributed and with medians and interquartile ranges (IQRs) if not. Categorical data were reported as absolute numbers and proportions.

Interpatient variability of drug levels at baseline and at both baseline and follow-up, was reported as median, 10 ^th^ to 90 ^th^ percentiles, and range. We determined the overall intrapatient variability of drug levels in those patients for whom a second drug level was available. In this cohort, we estimated the intrapatient geometric coefficient of variation (gCV _intra_ ) of both trough and peak levels from estimates of within-subject variance obtained from log-transformed data, and reported their 95% confidence intervals (CIs) using the methods described by Bland and Altman. 26 27 28

To assess whether a single drug level measurement is predictive of sustained high apixaban levels in patients treated with 5-mg BID apixaban, we chose the 80 ^th^ percentiles of baseline levels (for both trough and peak) to classify such patients with a high drug level. This was based on the following two considerations: (1) halving the dose of apixaban would result in approximately the corresponding 50% reduction in drug level; 6 29 and (2) if drug levels above the upper quintile were halved, the resultant level would be expected to fall in the third quintile of levels and unlikely to be too low to increase the risk of thrombosis. 30 We reported the proportions (and their 95% CIs using the Wilson Score Interval) 31 of patients whose levels remained in the upper extreme quintile.

We performed four additional analyses. First, we repeated our analysis on identifying patients on standard dose apixaban with persistently high levels but instead examined whether values at the second visit predicted those at baseline. Second, we estimated the proportional difference in levels between both visits for those with an initial level in the upper quintiles, and third, determined the proportion of patients within this subgroup who had a subsequent level that fell below the median. Fourth, we further examined for consistency by repeating all analyses on intrapatient variability while excluding the patients with VTE.

All analyses were performed with R (version 4.0.5 or higher) within RStudio (version 1.4.1106), 32 33 or Microsoft Excel for Microsoft 365 (version 16.0.13901.20436). 34

## Results

### Patient Selection

**Fig. 1:**
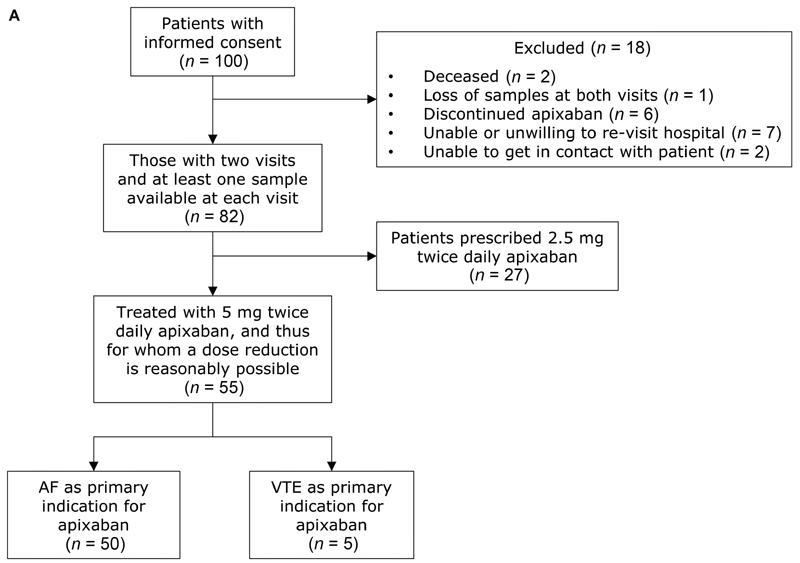
Patient selection: this figure summarizes the follow-up of recruited patients, and the reasons for exclusion from the final dataset. AF, atrial fibrillation; VTE, venous thromboembolism.

Fig. 1 summarizes the follow-up of recruited patients and the reasons for exclusion. We enrolled a total of 100 patients of whom 82 came for a second visit and had at least one sample available at each visit.

### Clinical Characteristics

Table 1 summarizes the clinical characteristics of patients. Of the 82 included patients, 55 (67.1%) were treated with 5-mg BID apixaban of whom 50 had AF as their primary indication for apixaban. Fourteen (28.0%) of these 50 patients had one ABC criterion, and only one patient (2.0%) received the standard dose off-label.

The mean (± SD) weight of the 55 patients treated with the standard dose was 84.5 (± 15.5) kg, and the median (IQR) age, body mass index, and creatinine clearance was 72.4 (66.5–78.7) years, 28.4 (26.0–31.7) kg/m ^2^ , and 82.5 (59.9–100.6) mL/min, respectively. Nine (16.4%) patients were concomitantly treated with a P-glycoprotein (P-gp) and/or CYP P450 3A4 (CYP3A4) inhibitor: six (10.9%) with amiodarone and three (5.5%) with diltiazem, all of whom had AF. The characteristics of the patients who provided informed consent but were excluded from all analyses were comparable to those who were included ( Supplementary Table S2 ).

### Interpatient Variability

Blood for trough levels was collected at a mean (± SD) of 13 hours and 28 minutes (± 1 hour and 53 minutes) after the last ingested dose, while blood for peak levels was collected at 2 hours and 58 minutes (± 10 minutes) after morning apixaban administration.

**Fig. 2:**
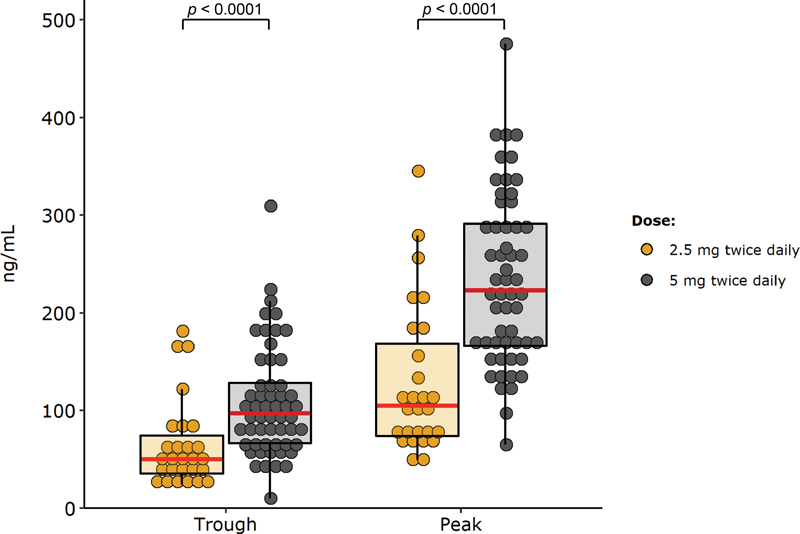
Inter-patient variability of apixaban: this graph illustrates the distribution of the initial drug level measurements in the 82 patients who came for a second visit. The horizontal red lines indicate the medians, the box the interquartile ranges, and the whiskers the first or third quartiles ± 1.5 times the interquartile ranges. The *p* -values were estimated using the Mann–Whitney test.

The distribution of initial drug levels in the 82 patients who came to both visits are presented in Fig. 2 , and that of levels as measured in the same cohort but when considering both the initial and second visit in Supplementary Fig. S1 . The median (10 ^th^ —90 ^th^ percentiles and minimum–maximum) of the initial measurements was 82.0 (36.4–178.9 and 10.0–309.0) ng/mL for trough levels, and 182.0 (74.6–336.6 and 33.0–411.0) ng/mL for peak levels. Patients prescribed the 5-mg BID apixaban had a significantly higher median trough (97.0 vs. 50.0 ng/mL; *p* < 0.0001) and peak level (223.0 vs. 105.0 ng/mL; *p* < 0.0001) than those prescribed the 2.5-mg BID dose. For patients treated with the standard dose who had a baseline level in the upper quintile (classified as “high”), the median (10 ^th^ —90 ^th^ percentile and minimum–maximum) level was 181.0 (149.8–220.4 and 117.0–309.0) and 348.9 (296.7–384.0 and 289.0–475.0) ng/mL for trough and peak levels, respectively.

### Intrapatient Variability

#### *Coefficient of Intrapatient Variation:*

The gCV _intra_ for trough and peak levels was 33.3% (95% CI: 27.6–39.3%) and 28.2% (95% CI: 23.4–33.2%), respectively. Patients prescribed 5-mg BID had a gCV _intra_ of 32.7% (95% CI: 25.9–39.9%) for trough levels and of 28.0% (95% CI: 22.2–34.0%) for peak levels. The values were similar when calculated only for the patients with AF (gCV _intra_ of 31.3% and 28.4% for trough and peak levels, respectively).

#### *Proportion of Patients Treated With 5-mg Twice Daily Apixaban With Sustained High Levels:*

**Fig. 3:**
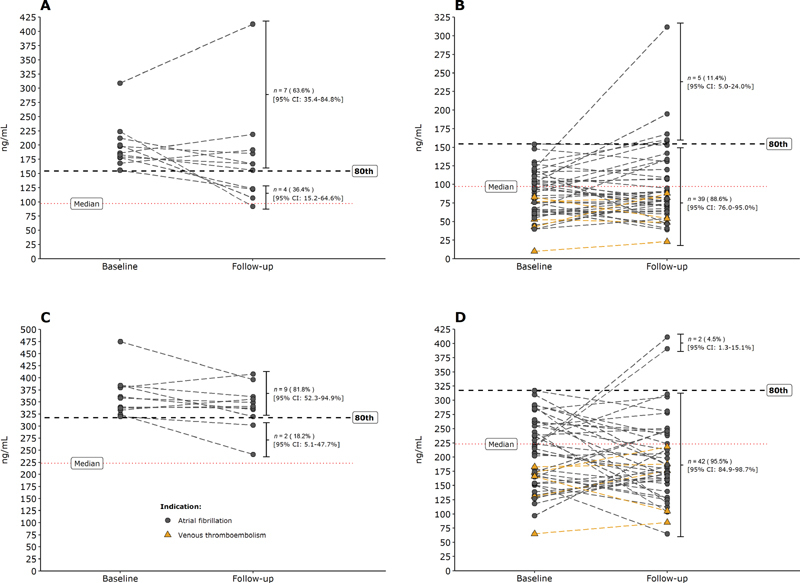
Intrapatient variability of apixaban in patients treated with the 5-mg twice daily dose, with an initial level in or below the upper quintile: these graphs illustrate the intra-patient variability of the 55 patients treated with 5-mg twice daily apixaban, with an initial trough ( **A and B** ) or peak level ( **C and D** ) in or below the upper quintile of levels. The cut-offs for the upper quintile at the first visit are 154.4 and 317.6 ng/mL for trough and peak levels, respectively. CI, confidence interval.

Fig. 3 shows that seven of 11 (63.6, 95% CI: 35.4–84.8%) patients receiving the standard dose who had baseline trough levels in the top quintile had a second level that remained in this quintile. The mean percent difference when comparing second with the initial drug level was −12.9% (range: −58.9% to +33.7%). Only one of 11 (9.1%, 95% CI: 1.6–37.7%) patients with a baseline high trough level had a subsequent level that dropped below the median. This patient had a trough level that dropped from 224 to 92 ng/mL with the second level being just below the median of 97 ng/mL.

The same figures also illustrates that nine of 11 (81.8%, 95% CI: 52.3–94.9%) patients receiving the standard dose apixaban with a baseline peak level in the upper quintile, had a second level that remained in this range. The mean percentage difference when comparing second with the initial drug level was −6.0% (range: −25.6% to +7.4%), and none had a repeated level that fell below the median.

For both peak and trough analyses, we observed similar results after excluding patients with VTE ( Supplementary Fig. S2 ). In general, patients taking 5-mg BID with sustained high peak or trough levels were more likely to have one or more ABC criteria (six of 10 patients, 60.0%) compared with those who did not have consecutive high levels (12 of 45 patients, 26.6%). In the 10 patients with sustained high levels, six (60%, 95% CI: 31.3–83.2%) had both sets of trough and peak levels (i.e., all four measurements) in the upper quintile; and of the 14 patients with an initial high level, eight (57.1%, 95% CI: 32.6–78.6%) had both the first trough and peak level in the upper quintile ( Supplementary Table S3 ).

#### *Measurements at the Second Visit to Predict Those at Baseline:*

Additional analyses that examined whether levels measured at the second visit predicted those measured at baseline produced similar results. Thus, six (54.5%, 95% CI: 28.1–78.7%) and nine (81.8%, 95% CI: 52.3–94.9%) of the 11 patients with their second level in the upper quintile had a preceding level that was also within the upper quintile ( Supplementary Fig. S3 ). Notably, regardless of the timing of measurement (i.e., trough or peak), at most, only one patient with a level in the upper quintile at follow-up, had a preceding level that crossed the median. Again, the results were consistent when we excluded the patients with VTE ( Supplementary Fig. S4 ).

## Discussion

We performed this study to determine whether a single apixaban measurement is predictive of sustained high drug level in patients who are treated BID with the 5-mg dose. Such information would allow physicians contemplating off-label dose reduction to select patients in whom dose reduction is less likely to lead to a thrombotic event. Our data indicate that such an approach has potential because most patients (approximately two-thirds or more) with an initial high trough and/or high peak level had a subsequent level that remained high when tested after 2 months, and in the remainder, none had a level that fell into the lowest quintile.

Although physicians who choose to implement off-label dose reduction have been criticized on the grounds that they risk exposing patients to thrombosis, such an approach might be justifiable in some patients with AF for the reasons mentioned hereinafter. First, many patients with AF who are at high risk of bleeding were underrepresented in the randomized trials. 9 10 Therefore, whether 5-mg BID is the optimal dose for these patients is unclear. 9 22 Second, there is consistent evidence that in patients with AF, there is a strong and continuous association between DOAC drug levels and bleeding, whereas the association with thrombosis is weaker. 4 30 35 36 37 Thus, results of randomized trials with dabigatran and edoxaban showed that, as drug levels rise, major bleeding risk steadily increases, whereas with decreasing levels, the risk of stroke does not increase substantially. 36 37 Information of a relationship between apixaban drug levels and clinical outcomes is limited to unpublished data from ARISTOTLE, a phase-III trial that compared apixaban with warfarin in patients with AF 3 and a published subgroup analysis of AVERROES, the phase-III trial that compared apixaban with aspirin in patients with AF deemed ineligible for treatment with vitamin-K antagonists. 30 Both studies showed that the risk of bleeding positively correlated with increasing drug levels but a relationship between levels and ischemic stroke was only observed in the patients enrolled in AVERROES for whom apixaban trough levels fell to within the lowest decile (i.e., ≤17 ng/mL). 4 30 So, although the data are limited, the findings are consistent among different DOACs and different patients treated with apixaban.

Since reducing the dose of apixaban from 5- to 2.5-mg BID results in a proportional reduction in drug levels, 4 6 21 29 patients with apixaban levels consistently in the upper quintile are unlikely to have levels that fall into the lower extreme when their dose is halved. Therefore, limiting off-label use of the 2.5-mg dose BID to patients whose drug levels are in the upper quintile is likely to mitigate against an increased risk of ischemic stroke.

Examples of patients likely to be at increased risk of high levels are those with at least one (extreme) ABC-criterion, and those concomitantly using one or more (dual) P-gp/CYP3A4 inhibitors. 4 6 16 38 39 Measuring a drug level in such patients or repeating the measurement in those developing incident risk factors for high drug exposure could complement current dose reduction strategy that are based on clinical factors only.

## Limitations

An important limitation of our study is its small sample size. However, it is reassuring that the drug levels in our cohort are consistent with those seen in patients enrolled in other observational studies and phase I to III clinical trials. 23 29 38 The main limitation of our study is that it was not performed in the population of interest, that is, patients who are treated with the standard dose of apixaban in whom off-label use of the reduced dose is contemplated.

## Conclusion

The trough and peak levels of apixaban in patients who have a high level on a single occasion, usually remain high when the assay is repeated in 2 months. Accordingly, the finding of a high apixaban level in patients deemed to be at high risk of bleeding, allows physicians contemplating off-label use of the 2.5-mg BID dose, to limit its use to selected patients who are less likely to be exposed to an increased risk of thrombosis.
