# Supplementary material for: Can a Single Measurement of Apixaban Levels Identify Patients at Risk of Overexposure? A Prospective Cohort Study
Source: TH Open. 2022 Jan 24;6(1):e10–7. doi: 10.1055/s-0041-1740492 (PMC8786560; doi:10.1055/s-0041-1740492)
Supplement: Supplementary file 1 — Supplementary Material [file 10-1055-s-0041-1740492-s210049.pdf]

Supplementary Information

Can a single measurement of apixaban levels identify patients at risk of overexposure? A prospective cohort study

Tim A.C. de Vries et al

October 20, 2021

Table of Contents

Table S1. STROBE Statement—Checklist of items that should be included in reports of cohort studies 3

Table S2. Patient characteristics of all eligible, excluded and included patients 5

Figure S1. Inter-patient variability of apixaban when considering both the initial and second level 6

Figure S2. Sensitivity analysis: Intra-patient variability of apixaban in patients with atrial fibrillation treated with the 5 mg twice daily dose 7

Trough levels: 7

Peak levels: 8

Table S3. Patient characteristics of patients on 5 mg twice daily apixaban by (sustained) high drug level status 9

Figure S3. Sensitivity analysis: Using the second measurement to classify patients with sustained high levels 10

Trough levels: 10

Peak levels: 11

Figure S4. Sensitivity analysis: Using the second measurement to classify sustained high levels in patients with atrial fibrillation 12

Trough levels: 12

Peak levels: 13

Table S1. STROBE Statement—Checklist of items that should be included in reports of cohort studies

	Item No	Recommendation	Check
**Title and abstract**	1	(*a*) Indicate the study's design with a commonly used term in the title or the abstract	✔
		(*b*) Provide in the abstract an informative and balanced summary of what was done and what was found	✔
**Introduction**			
Background/ rationale	2	Explain the scientific background and rationale for the investigation being reported	✔
Objectives	3	State specific objectives, including any prespecified hypotheses	✔
**Methods**			
Study design	4	Present key elements of study design early in the paper	✔
Setting	5	Describe the setting, locations, and relevant dates, including periods of recruitment, exposure, follow-up, and data collection	✔
Participants	6	(*a*) Give the eligibility criteria, and the sources and methods of selection of participants. Describe methods of follow-up	✔
		(*b*) For matched studies, give matching criteria and number of exposed and unexposed	NA
Variables	7	Clearly define all outcomes, exposures, predictors, potential confounders, and effect modifiers. Give diagnostic criteria, if applicable	✔
Data sources/ measurement	8[a]	For each variable of interest, give sources of data and details of methods of assessment (measurement). Describe comparability of assessment methods if there is more than one group	✔
Bias	9	Describe any efforts to address potential sources of bias	✔
Study size	10	Explain how the study size was arrived at	✔
Quantitative variables	11	Explain how quantitative variables were handled in the analyses. If applicable, describe which groupings were chosen and why	✔
Statistical methods	12	(*a*) Describe all statistical methods, including those used to control for confounding	✔
		(*b*) Describe any methods used to examine subgroups and interactions	✔
		(*c*) Explain how missing data were addressed	✔[b]
		(*d*) If applicable, explain how loss to follow-up was addressed	✔
		(*e*) Describe any sensitivity analyses	✔
**Results**			
Participants	13[a]	(a) Report numbers of individuals at each stage of study—e.g. numbers potentially eligible, examined for eligibility, confirmed eligible, included in the study, completing follow-up, and analyzed	✔

	Item No	Recommendation	Check
		(b) Give reasons for non-participation at each stage	✔
		(c) Consider use of a flow diagram	✔
Descriptive data	14[a]	(a) Give characteristics of study participants (e.g. demographic, clinical, social) and information on exposures and potential confounders	✔
		(b) Indicate number of participants with missing data for each variable of interest	✔[b]
		(c) Summarize follow-up time (e.g., average and total amount)	✔
Outcome data	15[a]	Report numbers of outcome events or summary measures over time	✔
Main results	16	(*a*) Give unadjusted estimates and, if applicable, confounder-adjusted estimates and their precision (e.g., 95% confidence interval). Make clear which confounders were adjusted for and why they were included	✔
		(*b*) Report category boundaries when continuous variables were categorized	✔
		(*c*) If relevant, consider translating estimates of relative risk into absolute risk for a meaningful time period	NA
Other analyses	17	Report other analyses done—e.g. analyses of subgroups and interactions, and sensitivity analyses	✔
**Discussion**			
Key results	18	Summarize key results with reference to study objectives	✔
Limitations	19	Discuss limitations of the study, taking into account sources of potential bias or imprecision. Discuss both direction and magnitude of any potential bias	✔
Interpretation	20	Give a cautious overall interpretation of results considering objectives, limitations, multiplicity of analyses, results from similar studies, and other relevant evidence	✔
Generalizability	21	Discuss the generalizability (external validity) of the study results	✔
**Other information**			
Funding	22	Give the source of funding and the role of the funders for the present study and, if applicable, for the original study on which the present article is based	✔

NA Not Applicable

[a] Give information separately for exposed and unexposed groups.

[b] Patients with incomplete longitudinal sets of drug level measurements were excluded from the final dataset. None of the reported patient characteristics had missing data.

Table S2. Patient characteristics of all eligible, excluded and included patients

	All (n=100)	Excluded (n=18)	Included (n=82)
Apixaban dose			
- 5 mg BID	68 (68.0)	13 (72.2)	55 (67.1)
- 2.5 mg BID	32 (32.0)	5 (27.8)	27 (32.9)
Indication			
- Atrial fibrillation	84 (84.0)	15 (83.3)	69 (84.1)
- Venous thromboembolism	16 (16.0)	3 (16.7)	13 (15.9)
Number of ABC criteria			
- 0	68 (68.0)	11 (61.1)	57 (69.5)
- 1	28 (28.0)	7 (39.9)	21 (25.6)
- ≥2	4 (4.0)	0 (0.0)	4 (4.9)
Age[a] - years	73.0 (66.3-79.7)	73.0 (67.1-77.6)	73.1 (66.1-79.8)
Male	59 (59.0)	13 (72.2)	46 (56.1)
Weight[a] – kg	82.0 (71.8-96.3)	81.3 (75.0-101.5)	82.0 (71.4-95.4)
BMI[a] – kg/m^2	28.2 (25.6-31.5)	28.0 (25.6-30.8)	28.3 (25.6-31.5)
CrCl[a] – ml/min	79.1 (60.2-101.5)	74.3 (51.9-110.6)	82.8 (61.1-101.2)
History of heart failure	19 (19.0)	4 (22.2)	15 (18.3)
Alcohol use	26 (26.0)	5 (27.8)	21 (25.6)
Ongoing smoking habit	5 (5.0)	1 (5.6)	4 (4.9)
P-gp and/or CYP3A4 inhibitor	14 (14.0)	3 (16.7)	11 (13.4)
- Amiodarone	8 (8.0)	2 (11.1)	6 (7.3)
- Diltiazem	5 (5.0)	0 (0.0)	5 (6.1)
- Ketoconazole	1 (1.0)	1 (5.6)	0 (0.0)
P-gp and/or CYP3A4 inducer	0 (0.0)	0 (0.0)	0 (0.0)

Continuous data are reported as mean±standard deviation if normally distributed, and as median (interquartile range)[a] if not. Categorical data are in number (percentage).

BID twice daily, *BMI* body mass index, *CrCl* Creatinine Clearance, *CYP3A4* Cytochrome P3A4 *CYP450*, *P-gp* P-glycoprotein.

Figure S1. Inter-patient variability of apixaban when considering both the initial and second level

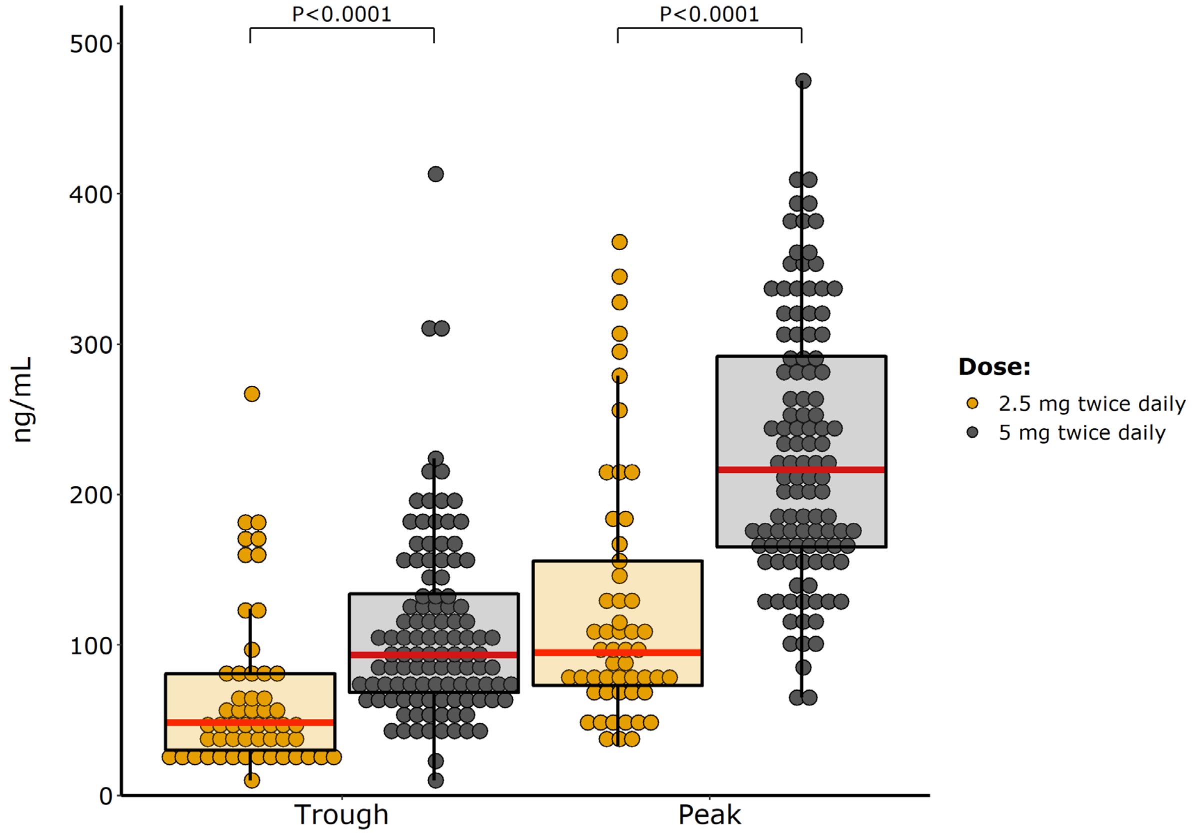

This graph illustrates the distribution of both the initial and second drug level measurements combined in the 82 patients who came for a second visit. The horizontal red lines indicate the medians, the box the interquartile ranges, and the whiskers the first or third quartiles±1.5 times the interquartile ranges. The P-values were estimated using the Mann-Whitney test.

The median (10th-90th percentiles, min-max) of the combined measurements was 81.0 (30.9-178.7, 10.0-413.0) ng/ml for trough levels, and 179.0 (74.0-338.6, 33.0-475.0) ng/ml for peak levels. Patients prescribed the 5 mg BID dose had a significantly higher median trough (93.5 vs. 66.5 ng/ml; p<0.0001) and peak level (216.5 vs. 95.0 ng/ml; P<0.0001) than those prescribed the 2.5 mg dose.

Figure S2. Sensitivity analysis: Intra-patient variability of apixaban in patients with atrial fibrillation treated with the 5 mg twice daily dose

<u>Trough levels:</u>

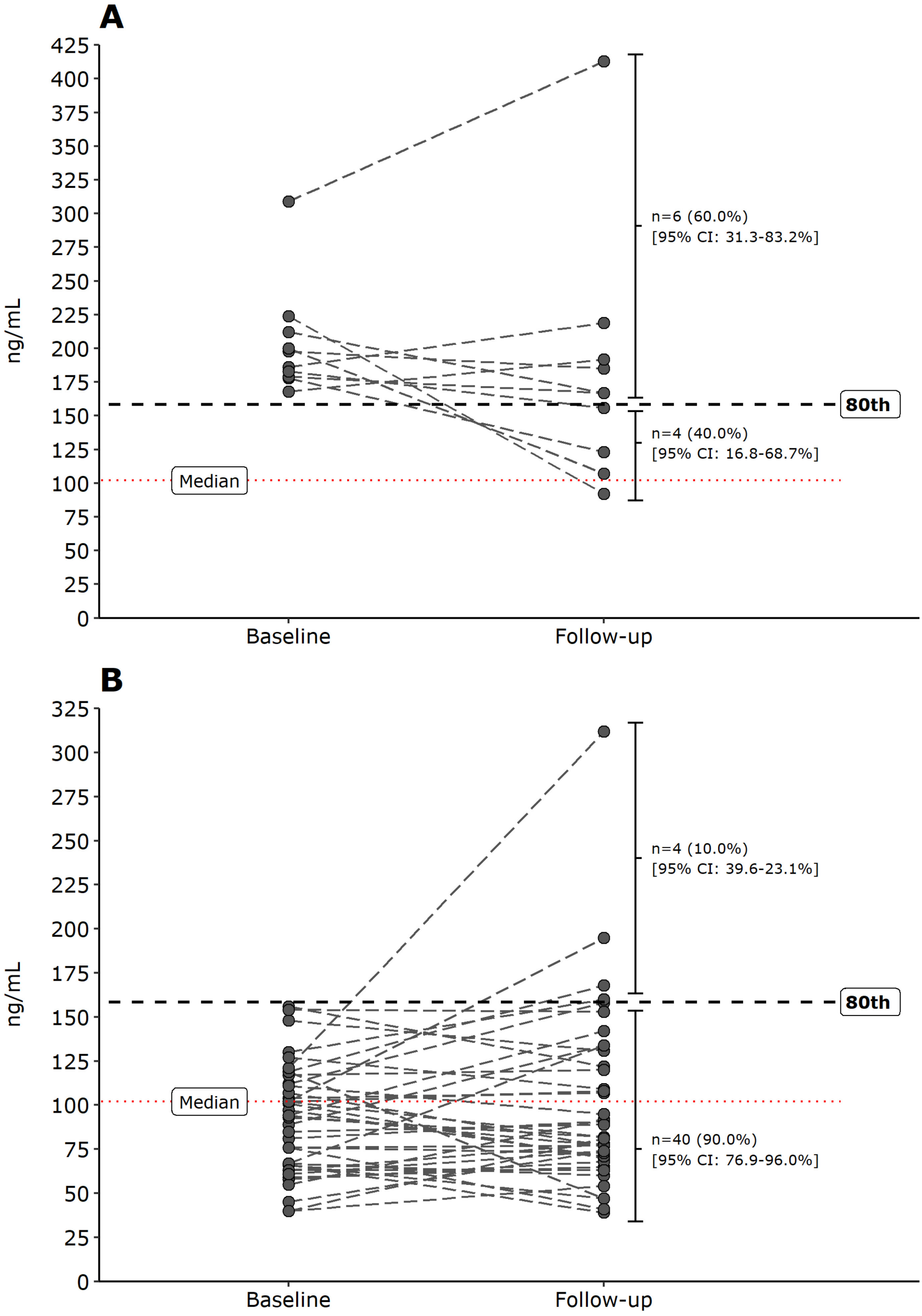

Peak levels:

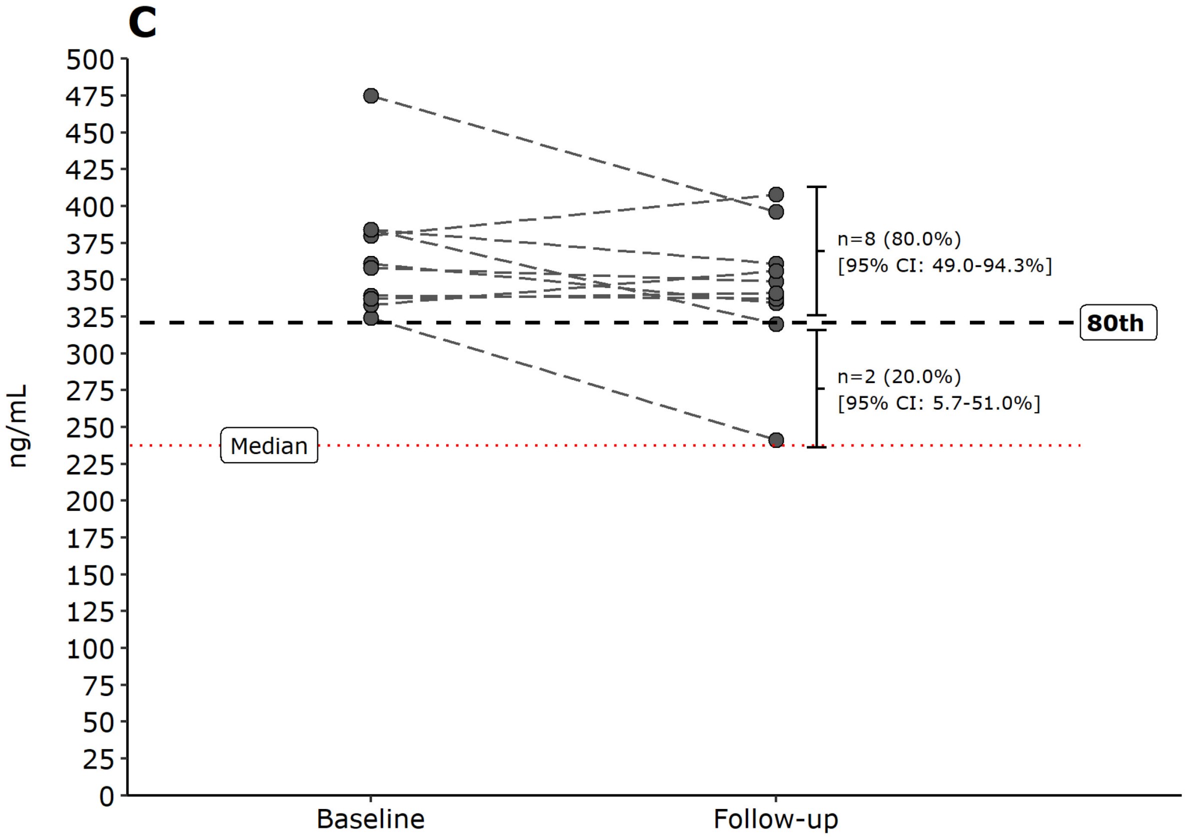

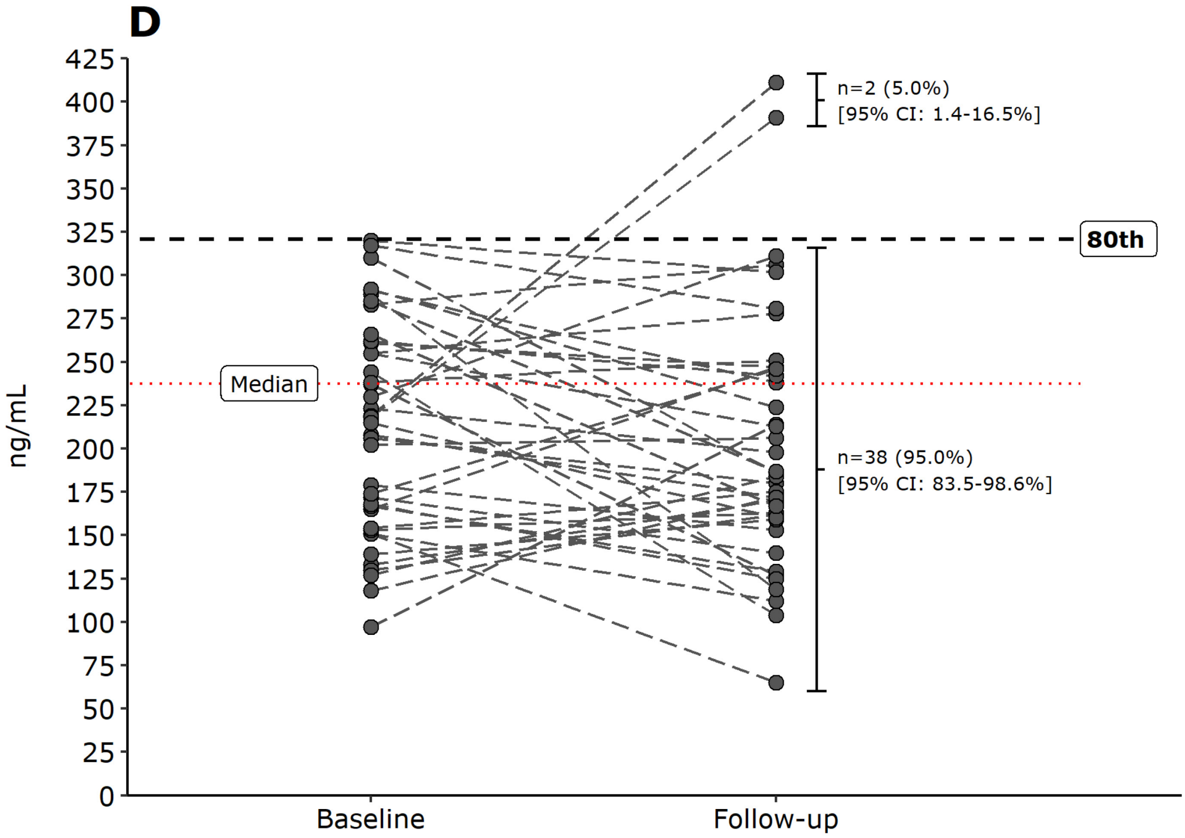

These graphs illustrate the intra-patient variability of the 50 patients with atrial fibrillation treated with 5 mg twice daily apixaban, and an initial trough (A and B) or peak level (C and D) in or below the upper quintile. The cut-offs for the upper quintile of levels at the first visit is 158.4 ng/ml and 320.8 ng/ml for trough and peak levels, respectively.

Table S3. Patient characteristics of patients on 5 mg twice daily apixaban by (sustained) high drug level status

	Sustained high levels[b]		High level[b] at baseline	
	**Yes (n=10)**	**No (n=45)**	**Yes (n=14)**	**No (n=41)**
Timing of measurement in respective subgroup				
- Both trough and peak	6 (60.0)	36 (80.0)	8 (57.1)	41 (100.0)
- Only trough	1 (10.0)	3 (6.7)	3 (21.4)	0 (0.0)
- Only peak	3 (30.0)	6 (13.3)	3 (21.4)	0 (0.0)
Indication				
- Atrial fibrillation	10 (100.0)	40 (88.9)	14 (100.0)	36 (87.8)
- Venous thromboembolism	0 (0.0)	5 (11.1)	0 (0.0)	5 (12.2)
Number of ABC criteria				
- 0	4 (40.0)	33 (73.3)	8 (57.1)	31 (75.6)
- 1	6 (60.0)	11 (24.4)	6 (42.9)	9 (22.0)
- ≥2	0 (0.0)	1 (2.2)	0 (0.0)	1 (2.4)
Age[a] - years	77.8 (71.3-81.5)	71.5 (66.0-78.8)	74.7 (69.4-79.7)	72.4 (66.0-77.5)
Male	5 (50.0)	29 (64.4)	8 (57.1)	27 (65.9)
Weight – kg	79.9±16.9	85.2±15.2	83.8±17.0	84.7±15.1
BMI[a] – kg/m^2	28.1 (27.5-30.1)	28.7 (25.8-33.0)	29.1 (27.6-31.4)	28.9 (25.3-31.5)
CrCl[a] – ml/min	62.4 (42.3-85.8)	84.4 (62.0-101.6)	76.1 (47.5-90.2)	86.4 (61.1-101.6)
History of heart failure	4 (40.0)	6 (13.3)	4 (28.6)	7 (17.1)
Alcohol use	3 (30.0)	13 (28.9)	5 (35.7)	11 (26.8)
Ongoing smoking habit	0 (0.0)	1 (2.2)	0 (0.0)	1 (2.4)
P-gp and/or CYP3A4 inhibitor	2 (20.0)	6 (13.3)	2 (14.3)	7 (17.1)
- Amiodarone	2 (20.0)	3 (6.7)	2 (14.3)	4 (9.8)
- Diltiazem	0 (0.0)	3 (6.7)	0 (0.0)	3 (7.3)
P-gp and/or CYP3A4 inducer	0 (0.0)	0 (0.0)	0 (0.0)	0 (0.0)

Continuous data are reported as mean±standard deviation if normally distributed, and as median (interquartile range)[a] if not. Categorical data are in number (percentage).

BMI body mass index, *CrCl* Creatinine Clearance, *CYP3A4* Cytochrome P3A4 *CYP450*, *P-gp* P-glycoprotein.

[b] The cut-offs to determine 'high levels' were 154.4 and 317.6 ng/ml for trough and peak levels, respectively, and were based on the 80th percentile of levels as determined at baseline in patients treated with 5 mg twice daily apixaban.

Figure S3. Sensitivity analysis: Using the second measurement to classify patients with sustained high levels

Trough levels:

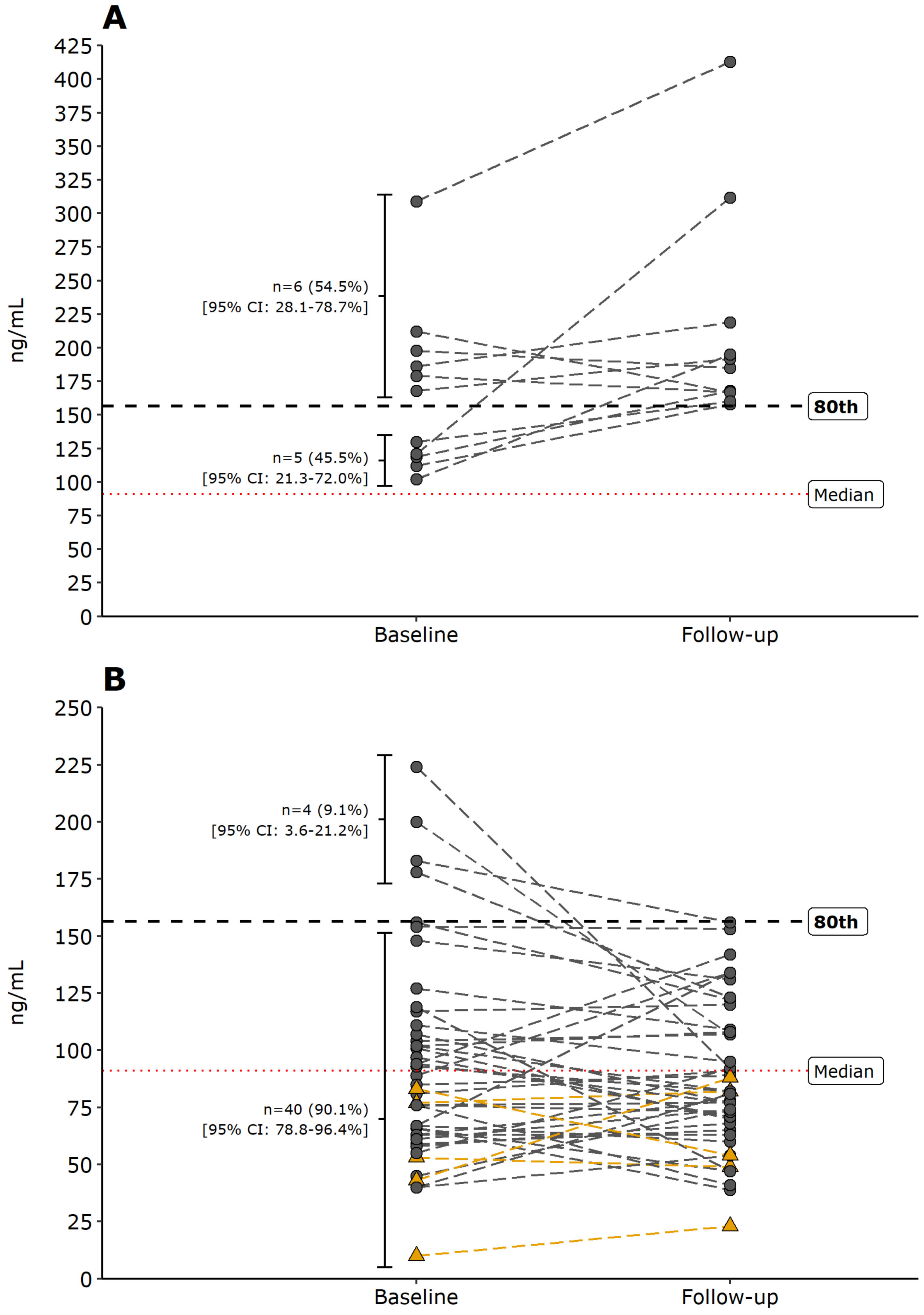

Peak levels:

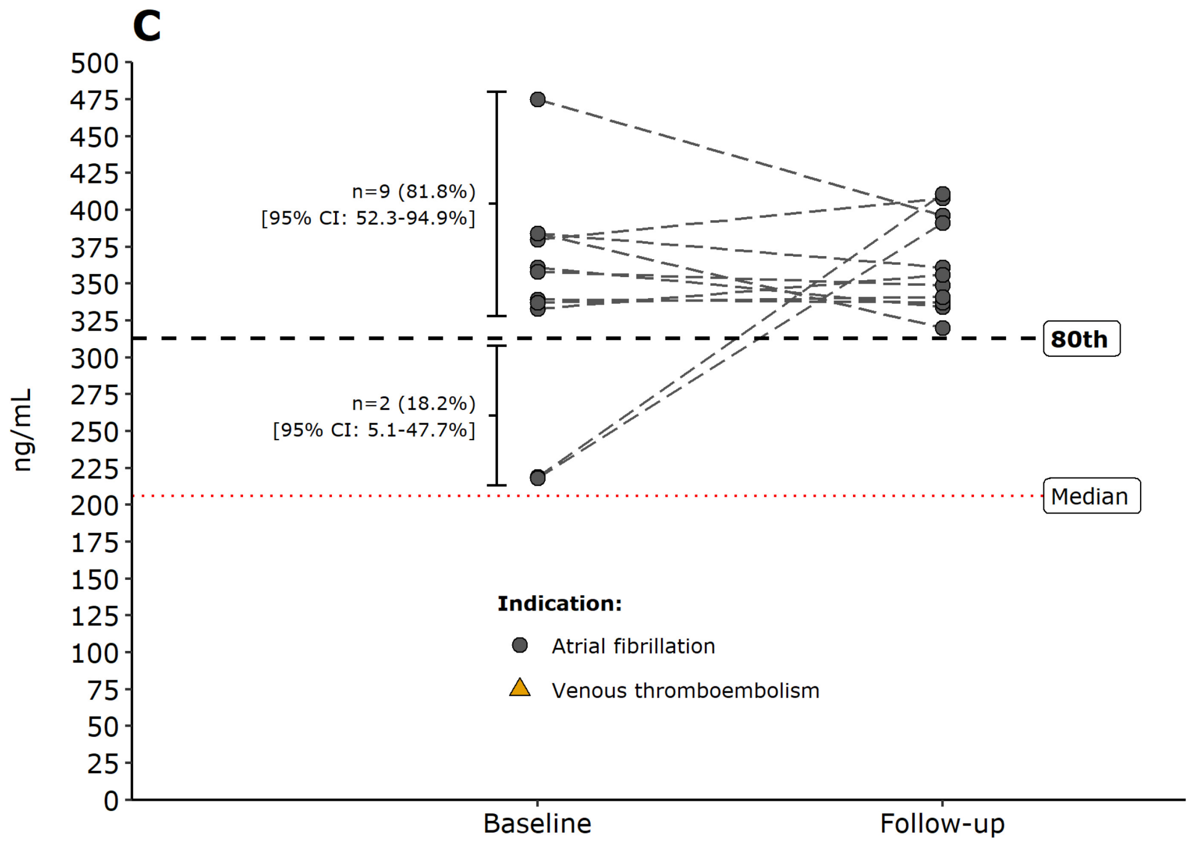

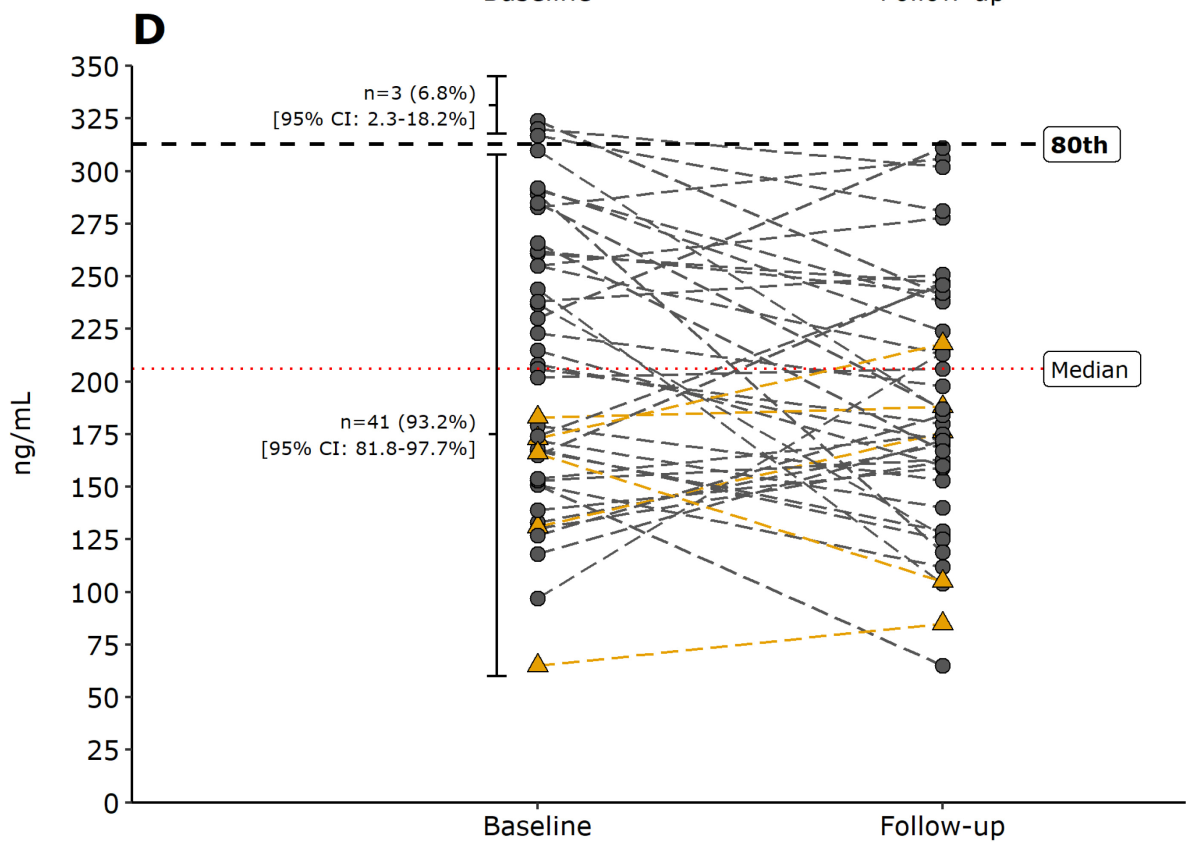

These graphs illustrate the intra-patient variability of the 55 patients with a trough (A and B) or peak level (C and D) at the second visit in or below the upper quintile of levels. The cut-offs for the upper quintile at the second visit is 156.4 ng/ml and 312.8 ng/ml for trough and peak levels, respectively.

Figure S4. Sensitivity analysis: Using the second measurement to classify sustained high levels in patients with atrial fibrillation

<u>Trough levels:</u>

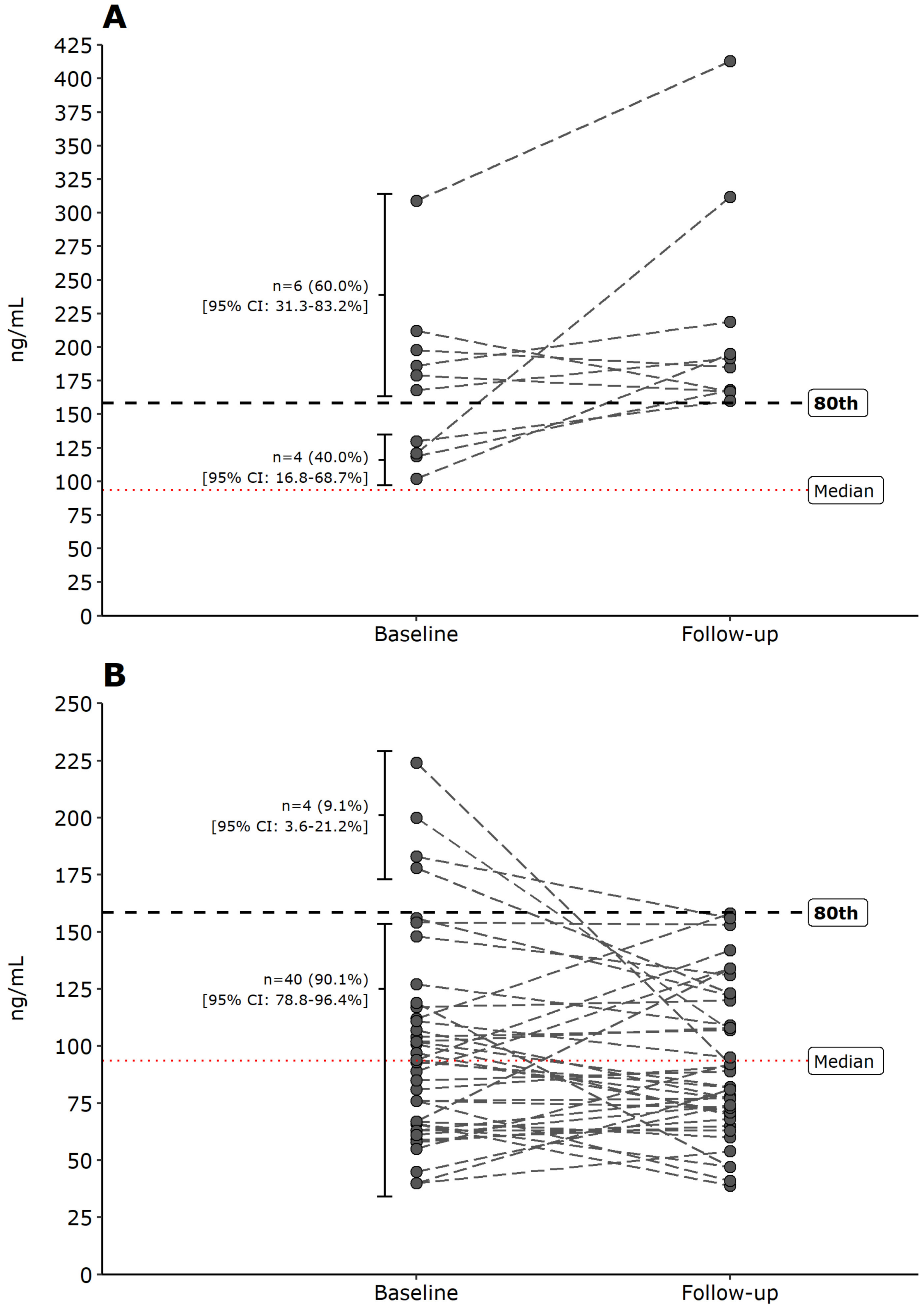

Peak levels:

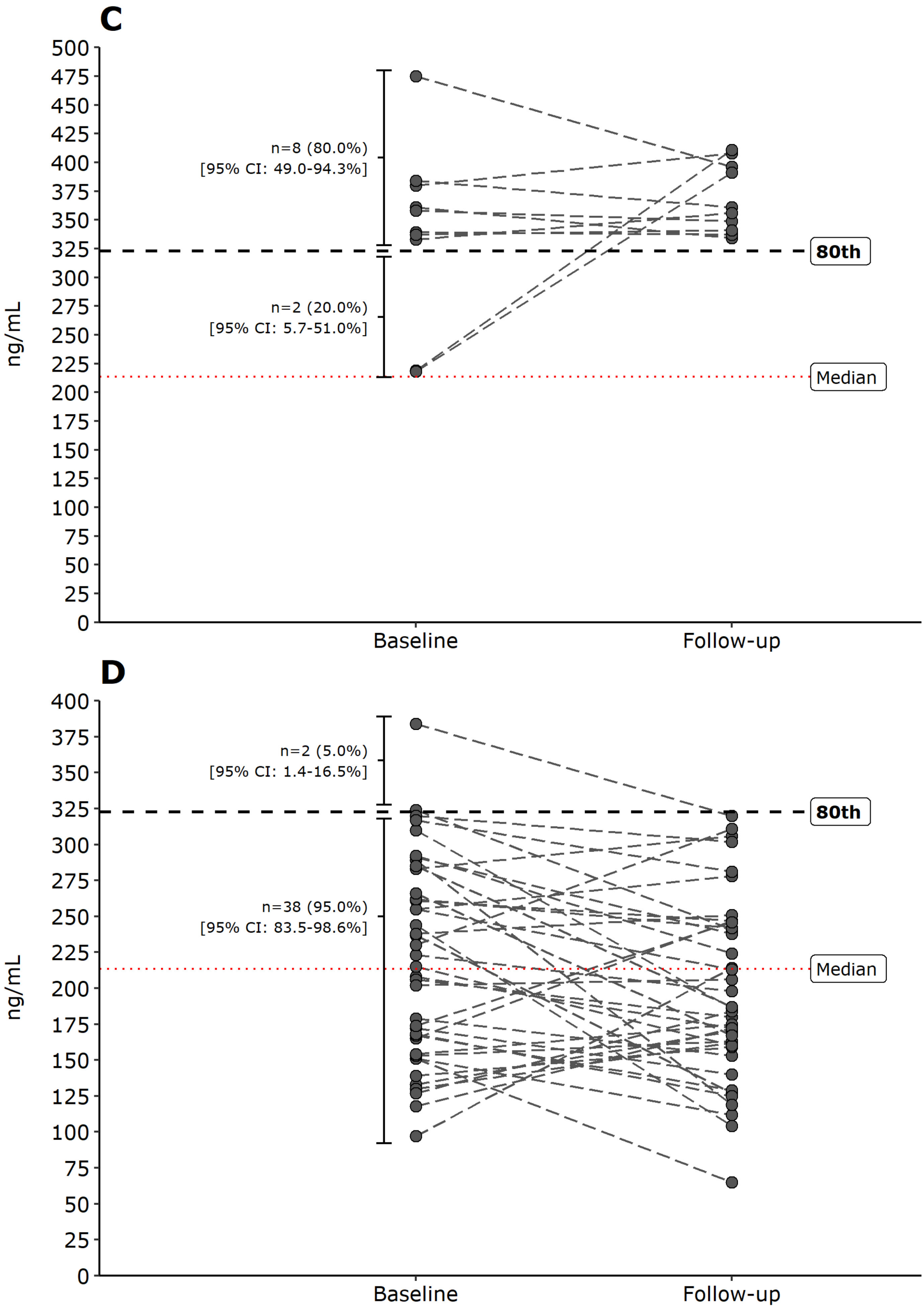

These graphs illustrate the intra-patient variability of the 50 patients with atrial fibrillation treated with 5 mg twice daily apixaban, and a trough (A and B) or peak level (C and D) at the second visit in or below the upper quintile of levels. The cut-offs for the upper quintile at the second visit is 158.4 ng/ml and 322.8 ng/ml for trough and peak levels, respectively.
